# Flexible echolocation behavior of trawling bats during approach of continuous or transient prey cues

**DOI:** 10.3389/fphys.2013.00096

**Published:** 2013-05-09

**Authors:** Kirstin Übernickel, Marco Tschapka, Elisabeth K. V. Kalko

**Affiliations:** ^1^Institute of Experimental Ecology, University of UlmUlm, Germany; ^2^Smithsonian Tropical Research InstituteBalboa, Panamá, República de Panamá

**Keywords:** approach, prey capture, water surface, reaction time, plasticity, *Noctilio leporinus*

## Abstract

Trawling bats use echolocation not only to detect and classify acoustically continuous cues originated from insects at and above water surfaces, but also to detect small water-dwelling prey items breaking the water surface for a very short time, producing only transient cues to be perceived acoustically. Generally, bats need to adjust their echolocation behavior to the specific task on hand, and because of the diversity of prey cues they use in hunting, trawling bats should be highly flexible in their echolocation behavior. We studied the adaptations in the behavior of *Noctilio leporinus* when approaching either a continuous cue or a transient cue that disappeared during the approach of the bat. Normally the bats reacted by dipping their feet in the water at the cue location. We found that the bats typically started to adapt their calling behavior at approximately 410 ms before prey contact in continuous cue trials, but were also able to adapt their approach behavior to stimuli onsets as short as 177 ms before contact, within a minimum reaction time of 50.9 ms in response to transient cues. In both tasks the approach phase ended between 32 and 53 ms before prey contact. Call emission always continued after the end of the approach phase until around prey contact. In some failed capture attempts, call emission did not cease at all after prey contact. Probably bats used spatial memory to dip at the original location of the transient cue after its disappearance. The duration of the pointed dips was significantly longer in transient cue trials than in continuous cue trials. Our results suggest that trawling bats possess the ability to modify their generally rather stereotyped echolocation behavior during approaches within very short reaction times depending on the sensory information available.

## Introduction

Bats that depend on echolocation to acquire food constantly adjust their echolocation calls to their surroundings and optimize call structure for increased information gain during detection, classification, and localization of prey (Schnitzler and Kalko, 2001). Especially bat species that use more than one prey capture mode in different habitats, e.g., aerial hawking and gleaning (*Myotis lucifugus* and *Myotis evotis*: Barclay, 1991) or from a water surface and in the air (*Myotis daubentonii*: Kalko and Schnitzler, 1989; *Noctilio leporinus*: Schnitzler et al., 1994) may need to adjust their echolocation and flight behavior extremely quickly, in response to the task on hand (Holderied et al., 2008).

A typical aerial hawking insect capture is a reaction to a continuous cue, i.e., to an object that a bat can lock its center of attention onto and home in on (e.g., Ghose et al., 2009; Surlykke et al., 2009; Moss and Surlykke, 2010). Such detection events are typically followed by modifications of echolocation behavior that are remarkably consistent: the animals switch from search mode to approach mode by decreasing pulse duration and pulse interval (e.g., Griffin, 1958; Simmons et al., 1979; Schnitzler et al., 1994; Kalko et al., 1998; Schnitzler and Kalko, 2001). The approach phase may be divided into an initial part and a terminal part. The latter is characterized by emission of usually one but sometimes two groups that are composed of many calls (Schnitzler and Kalko, 2001; Melcón et al., 2007). This terminal part is essential for continuously updating the information on the exact location of the prey and in most species may be subdivided into two components, final buzz I with successively shortening pulse intervals and final buzz II with very short but invariant pulse intervals (e.g., Kalko and Schnitzler, 1989; Siemers and Schnitzler, 2000; Melcón et al., 2007). The emission of echolocation calls typically ceases shortly before prey contact and is resumed after completion of the capture attempt (e.g., Schnitzler and Kalko, 2001).

Just as aerial hawking bats react to flying prey, trawling bats may perform stereotyped capture attempts upon detecting potential insect prey floating on the water surface that provides a continuously detectable acoustic cue. However, they have also developed the ability to forage on water-dwelling prey (e.g., fish, shrimp) (e.g., Brooke, 1994; Blood and Clark, 1998; Siemers et al., 2001; Aihartza et al., 2008). Water-dwelling prey may provide only temporary acoustic cues (hereafter: transient cues), i.e., a short disturbance that disappears within about 50–100 ms after breaking the water surface (Schnitzler et al., 1994). Trawling bats, such as the Greater Bulldog bat *N. leporinus*, recognize these stimuli as cues for prey and react with the emission of an approach phase and a capture attempt by dipping their feet near the center of the expanding ripples in the water (“pointed dips”, Schnitzler et al., 1994).

We hypothesized that bats with such flexible hunting behavior are likely to also possess adaptive plasticity in their echolocation behavior (e.g., Schnitzler and Kalko, 2001). Therefore, we presented *N. leporinus* with continuous and transient prey cues and tested whether and how the animals adapted their echolocation and flight behavior when approaching these targets. We compared the bats' behavior in both situations using ultrasound recordings with synchronized high-speed video.

We wanted to pinpoint a stable point in time for the onset of the approach phase in continuous cue trials, indicating the instant in time prior to prey contact that allows the bat to easily perform all necessary behavior in the remaining time prior to prey contact, similar to the wire avoidance task with *Myotis lucifugus* in Grinnell and Griffin (1958). Additionally, we wanted to assess a minimal reaction time between the onset of the transient stimulus and the onset of the approach phase and expected to find values around 50–60 ms similar to earlier reported minimal reaction times for e.g., *Myotis nattereri* and *Eptesicus fuscus* (Webster, 1967; Masters et al., 1985; Melcón et al., 2007).

We anticipated that the disappearance of the transient cue during the approach would have an effect on the echolocation behavior of the bat, because prey item localization is not possible anymore. To detect modifications of the approach behavior during the approach of a transient cue, we compared several call parameters between continuous and transient cue approaches, i.e., pulse intervals, pulse durations, and call composition (relation between duration of quasi-constant frequency (QCF) and frequency modulated (FM) components).

We expected the duration of the dip to be longer in transient cue trials than in continuous cue trials, due to the uncertainties the bats are facing when trying to grasp an undetectable prey item. Furthermore, we expected to find faster resumption of call emission after contact when the capture attempt has failed (Britton and Jones, 1999) so that the bat may achieve fast updates of information after a failed prey capture (Ghose et al., 2009).

Our results may provide a valuable contribution to the ongoing discussion of how quickly bats are able to adapt their echolocation behavior while approaching different cues and how they adjust their echolocation when a situation changes before completion of the approach.

## Materials and methods

### Study site and animals

We caught two male and one female *Noctilio leporinus* on Barro Colorado Island (BCI), a field station of the Smithsonian Tropical Research Institute (for details on the study area refer to Leigh, 1999) during two field stays between November 2009 and May 2010. After capture we allowed each bat to habituate for one night to the flight cage before we began task related training and experiments in the second night of captivity. We kept the bats individually and fed them with small fish, mealworms (larvae of *Tenebrio molitor*, Coleoptera), and occasionally locally caught bushcrickets. For supervision of nutritional status we monitored the weight of daily food intake ($\bar{x}$ = 24 g; range 15–32 g) as well as body weight of each bat. We released all bats in healthy condition with weight equal to the weight at capture ($\bar{x}$ = 57 g) or slightly increased ($\bar{x}$ = +0.7 g). All animals were released close to their capture site in the night after trial completion.

Permission of scientific collecting was provided by Autoridad Nacional del Ambiente (ANAM) and Ministerio de Desarrollo Agropecuario (MIDA). Our experiments complied with the national animal care policies (IACUC No. 2008-10-06-24-08).

### Experimental setup

**Figure 1:**
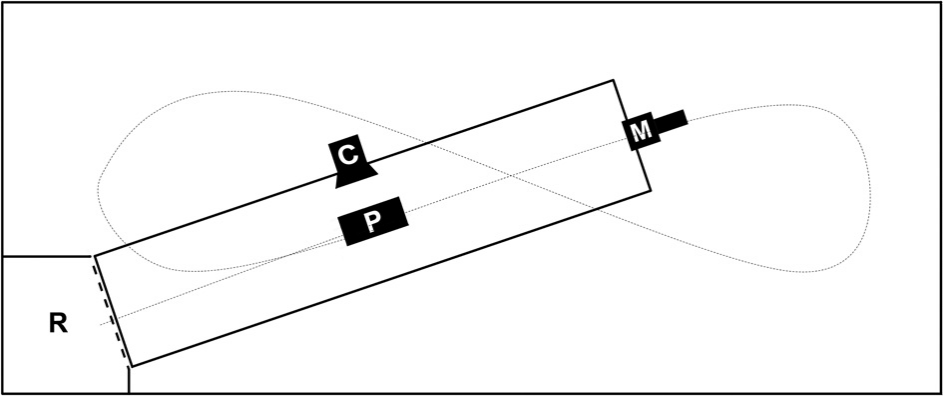
**Scheme of trial set up in the flight cage (12 × 5 × 2 m); C, high-speed camera filming the moment of prey capture; M, microphone position during all trials pointing over the length of the pond (7 × 1.5 m) toward the roost; P, prey area, varying in distance to roost (*d* = 3.5 ± 0.4 m); R, roost; short dashed line, curtain—raised during continuous cue trials; long dashed line, approximate trial trajectory of trained bats**.

We performed all experiments in a flight-cage (12 × 5 × 2 m) with an artificial pond (7 × 1.5 m) and a roost at one corner of the flight cage (Figure 1). The camera was positioned to film the instant of prey capture while the microphone was at the side of the pond opposite the perch and 40–50 cm above the water surface. In all tasks we offered prey objects that consisted of a piece of fish (weight $\bar{x}$ = 0.8 ± 0.3 g), mounted at varying distances from the perch ($\bar{x}$ = 3.5 ± 0.4 m) in order to avoid habituation of the bat to one single prey location and hence to increase the need for precise prey localization through echolocation. All three bats mastered the continuous cue task during the first night of training and learned within two more nights to approach also our transient cues.

We either presented a continuous or a transient cue at a time. In preparation for continuous stimulus trials, we lowered a curtain between the roost and the pond to prevent the bat from detecting the prey object prior to leaving the roost. First we placed the prey slightly protruding from the water surface, i.e., for 3–5 mm, then we raised the curtain. A trained bat would immediately leave the perch, fly 5–20 cm above the water surface in a straight line toward the prey and attempt capture (sample flight-path, Figure 1).

To provide the stimulus for the transient cue task, we used a device similar to the “artifish” used by Schnitzler et al. (1994). It consisted of a small plastic tube (Ø 4 mm) connected to a small air pump (LifeTech 3500) and a custom-made control device (scientific electronic workshop, University of Ulm, Germany). Upon being powered it produced small water splashes of $\bar{x}$ = 267 ± 75 ms duration in intervals of 3 s. Neither the free end of the tube nor the prey item mounted close to it protruded from the water surface. This guaranteed acoustical undetectability of the prey until the “artifish” produced a stimulus that broke the water surface. The position of the transient cue in the pond varied in the same area as the continuous cue. At the beginning of the transient cue trials we encouraged the bats to fly and they generally started immediately to search for prey objects at the water surface. While a bat was flying we activated the “artifish”. We triggered video and audio recordings whenever the bats reacted to the stimulus by dipping at the prey location while flying toward the microphone (Figure 1).

Whenever the bats dipped at the prey position, but lost the prey item while pulling it out of the water we scored this as a failed trial in both tasks.

### Data recording and analysis

We recorded the behavior of the bats with a high-speed video camera (CamRecord 600 × 2, Optronis, Kehl, Germany) set to a frame rate of 850 fps and a shutter time of 1/3000, using the manufacturers' software (Camcontrol V4.04, Optronis, Kehl, Germany). We recorded echolocation calls directly onto the hard disk of a laptop, using a condenser microphone (CM16/CMPA, Avisoft bioacoustics, Berlin, Germany) connected to a sound interface (116 Hm or 416 H, Avisoft bioacoustics) with a sampling rate of at least 300 kHz, using Avisoft software (Avisoft Recorder, version 3.3 to version 3.4.2, Avisoft bioacoustics, Berlin, Germany). Audio and video recordings were triggered synchronously by using a manual trigger device connected to both systems. We controlled both recording systems with the same laptop (Lenovo IBM 3000N200T8300 XP pro, Mainz, Germany).

We synchronized and analyzed audio and video data with custom-made software (Highsync, Version 0.94, Slomotec, Dr. Frank and Hella Gabler GbR, Frankfurt, Germany) and corrected for sound travel time to the microphone considering ambient temperature (recorded with a data-logger for temperature, humidity, and pressure, MSR Electronics GmbH, Model: 145, Henggart, Switzerland) and distance between camera position and microphone. For a detailed audio analysis we used SasLab Pro (version 5.2.06, Avisoft bioacoustics).

We tested two situations with two possible outcomes each: successful (1) and failed (2) continuous cue trials and successful (3) and failed (4) transient cue trials. 36 trials (2 tasks × 2 outcomes × 3 bats × 3 repetitions) entered our data analysis. For statistical comparisons we performed *t*-tests and Mann–Whitney-*U*-tests in SigmaStat 3.5 (Systat Software Inc., Chicago, IL 60606, USA), unless stated otherwise. Initially, we compared the selected parameters within one task across successful and failed trials. Only when these tests revealed no significant differences across trials in both tasks, the data per task were pooled to allow the use of the full dataset for comparison across tasks.

To enable comparisons across trials and tasks we required a reference point that allowed an alignment of all sequences. For this we used (a) the moment of contact between the bats' feet and prey in continuous cue trials and equivalently (b) the instant when the bats' feet passed the location of the water splash in transient cue trials. For all time-based analyses we defined these instants as zero and present all time information relative to this point of reference. Events occurring before the prey contact therefore scored negative time values.

For the acoustic analysis of echolocation calls we took into account that bats decrease the pulse amplitude successively throughout an approach (Hartley et al., 1989; Surlykke and Moss, 2000; Boonman and Jones, 2002). To compensate for systematic errors in measurements based on amplitude we normalized all calls (Holderied et al., 2008) to 75% of relative sound intensity. For measurements we used the automated measurement function of SasLab Pro set to a threshold of −40 dB relative to maximum amplitude and to measure peak frequencies at time intervals of 0.3 ms. We analyzed the audio data in a flat top spectrogram window, with an FFT length of 1024, 96.87% overlap, and a resulting reading accuracy of 293 Hz and 0.11 ms. We analyzed all echolocation calls emitted between −0.9 s before prey capture and ca. 0.3 s after prey capture. For each call we extracted pulse interval, pulse duration and the duration of QCF and FM components. We defined the moment of the switch between QCF and FM components within one call as the first of three 300 Hz intervals that were steadily declining in frequency.

The confined space of flight-cages generates increased pulse-echo overlap in comparison to field situations, and bats generally respond to this situation by using shorter calls during orientation flight (Suthers, 1965; Surlykke and Moss, 2000). We therefore obtained reference values of orientation flight in the cage by analyzing calls that were emitted between −0.9 s and −0.6 s before prey contact in all 36 continuous and transient cue trials and calculated for each individual the mean and standard deviations for pulse duration, pulse interval and QCF and FM components.

**Figure 2:**
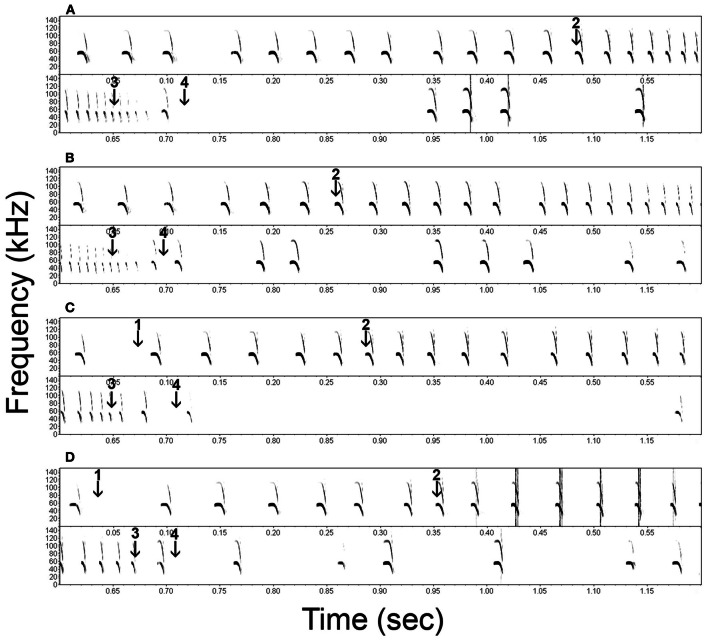
**Representative spectrograms of the echolocation behavior of *Noctilio leporinus* (bat 1) during the performance of a successful (A) and failed (B) approach to a continuous cue and successful (C) and failed (D) approaches to a transient cue.** Arrows indicate the onset of the transient stimulus (1), the onset of the approach phase (2), the end of the approach phase (3) and the instant of first prey contact (4).

**Figure 3:**
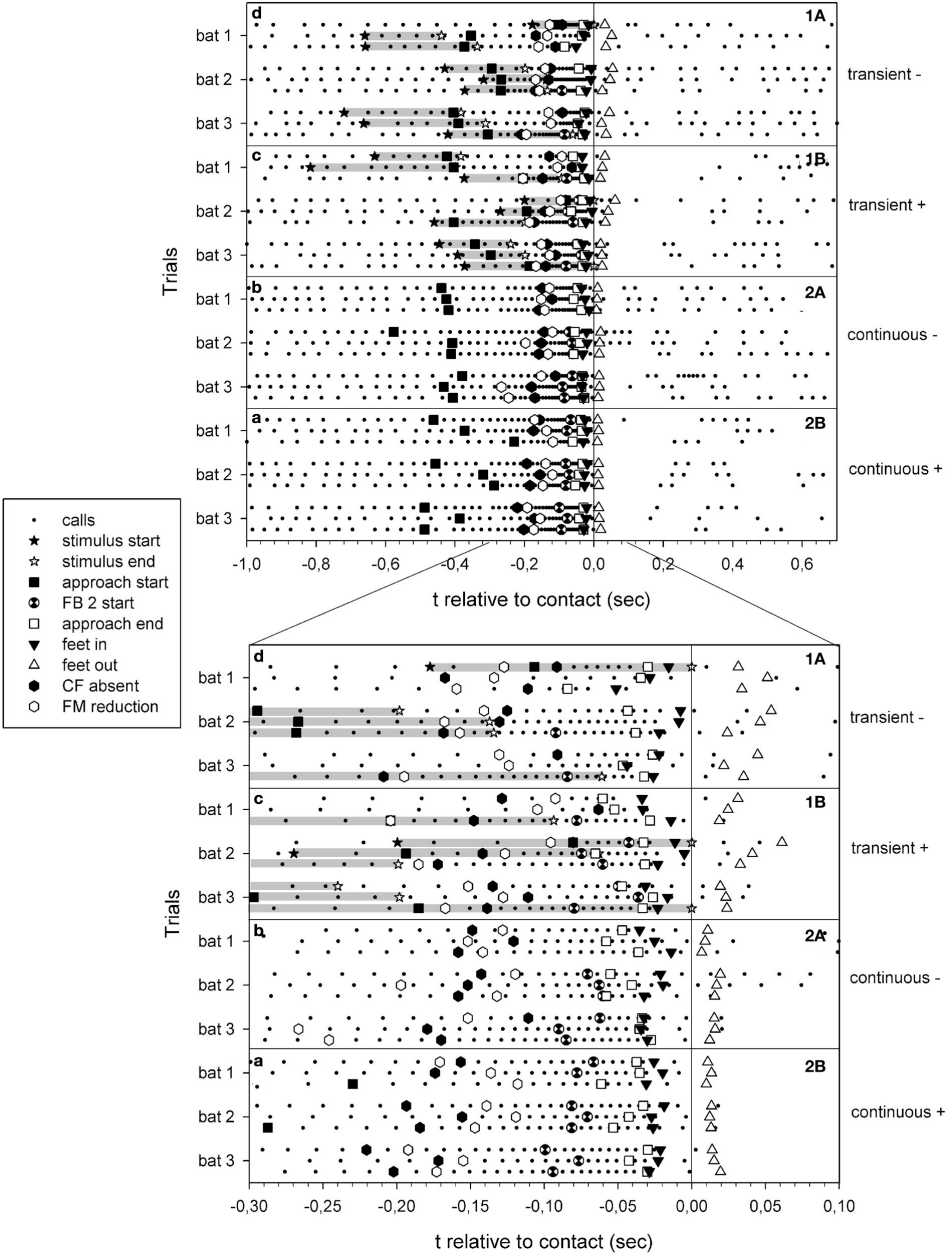
**Summary of echolocation phases of all selected trials of failed (A) and successful (B) approaches to the site of transient cues (1) and continuous cues (2).** The trials are presented as a function of time relative to prey contact/or water splash location arrival (zero). Each dot represents the onset of a call. Gray bars: duration of transient cues.

**Figure 4:**
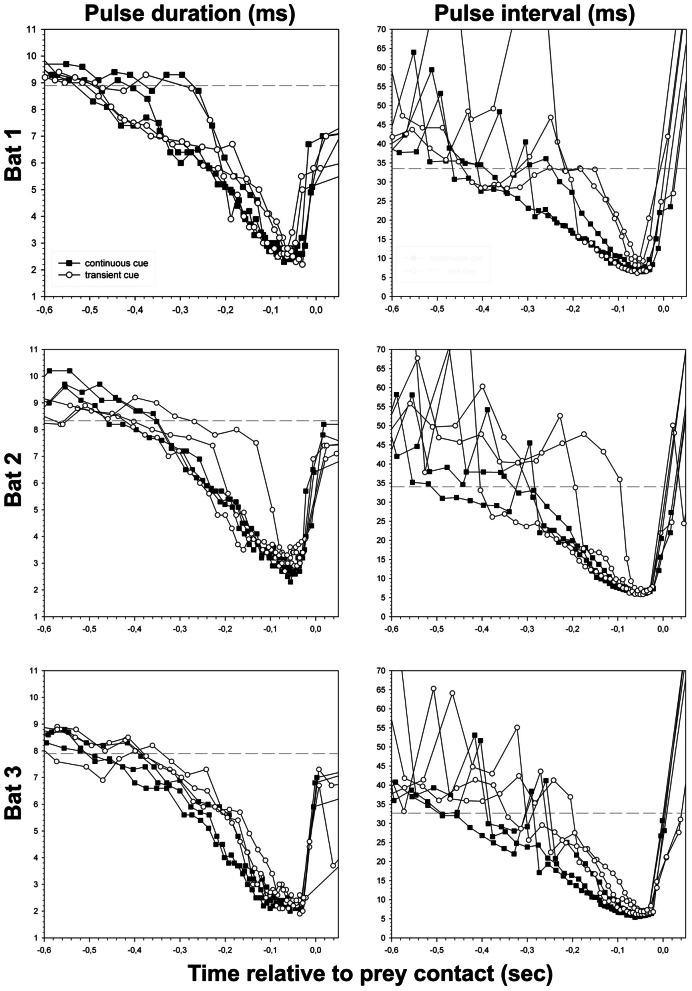
**Pulse duration and pulse interval of all successful approaches to the site of transient (unfilled circles) and continuous cues (filled squares), plotted separately for each bat.** Dashed lines indicate the threshold of approach phase onset calculated for each bat (see Table 1 and “Material and Methods” section).

We defined the onset of the approach phase per bat as the beginning of the first call that had shorter pulse duration and pulse interval than the previously determined reference values minus one standard deviation (Table 1, arrows 2 in Figure 2, dashed lines in Figure 4). We defined the end of the acoustic approach phase as being at the end of the shortest call in the sequence (arrows 3 in Figure 2, Figure 3) (Holderied et al., 2005). We defined final buzz II as existent in those trials where a minimum of two successive pulse intervals showed a pulse interval of <7 ms (Figure 3). For measuring the maximum sound pressure level emitted by *Noctilio leporinus* in the flight-cage, we used the same equipment in a similar set-up as described in Brinkløv et al. (2011).

**Table 1:** **Reference values for pulse duration (PD) and pulse interval (PI) for each bat during orientation phase**.

**Individual**	**PD mean (±*SD*)**	**PI mean (±*SD*)**
Bat 1	9.4 (±0.5)	52.0 (±18.9)
Bat 2	9.1 (±0.8)	49.5 (±15.5)
Bat 3	8.5 (±0.6)	51.2 (±18.5)

By definition, the approach phase in a trial began when both pulse duration and pulse interval fell below the threshold values calculated as mean value minus one standard deviation (SD).

### Comparison of behavior

We compared echolocation behavior of bats approaching transient cues to bats approaching continuous cues. We tested for significant differences in onset and end of the approach phase relative to prey contact across tasks.

For a better understanding of the timing of bats reacting to transient cues we also extracted from the video recordings the onset of the stimulus relative to prey contact in each trial and approximated the minimum reaction time by measuring the time intervals between the onset of a transient stimulus and the onset of the approach phase in the transient cue trials.

We analyzed and compared the changes in echolocation call components (QCF and FM) during the overall pulse duration reduction until the end of the approach phase. For this, we compared the timing of the first call without a QCF component within call sequences and we compared the onset of FM component reduction, defined as the instant when the FM component duration fell below mean duration in orientation flight minus one standard deviation ($\bar{x}$ = 4.6 ± 0.5 ms). To translate the changes in temporal echolocation behavior, as defined, into a distance to prey scale, we used video observations on flight speed of *N. leporinus* in the flight cage, to approximate the distances when the changes took place. Additionally, we tested if the presence or absence of final buzz II differed significantly across tasks (Chi-square-test) and we compared minimal pulse intervals across tasks. To investigate if the duration of the dip, i.e., the duration of contact between feet and water, differed across tasks, we calculated for each trial the time difference between instant of first contact between feet and water and the moment when the feet lost contact with the water, and compared this duration across tasks. Furthermore, we observed the degree to which the bats hit the exact location of the transient cue after it had already disappeared.

Calls occurring after the end of the approach phase were classified as post buzz calls type 1 and type 2. Type 1 calls had the same overall structure as final buzz II calls, and consisted only of a low intensity FM component that was longer in duration than the shortest call that defined the end of the approach phase. Type 2 consisted of a QCF and a FM component, mostly with higher intensity, similar to orientation phase calls in the flight cage. Either type 1 or type 2 calls, or a combination of both were always present shortly before, during, and/or shortly after prey contact. We documented the emission of post buzz calls type 1 and type 2 and how call continuity was related to success or failure of the capture attempts.

## Results

### Echolocation: orientation phase

Measurements of call parameters for the three individuals during the orientation phase ranged consistently about 9 ms for pulse durations and pulse intervals of 51 ms (Table 1). Duration of QCF components was 4.5 ms (median; quartiles 25%: 3.9 ms, 75%: 5.1 ms), and FM components lasted 4.5 ms (median; quartiles 25%: 4.2 ms, 75%: 4.8 ms). There was no significant difference between the durations of the two components (Wilkoxon signed rank test *W* = 1579.0; *P* = 0.32; *n* = 213).

### Approach of the prey cue

Analysis of differences across successful and failed trials within tasks revealed no significant differences. The onsets of transient stimuli varied between −817 ms and −177 ms before prey contact, but revealed no significant differences in the time interval between the onset of the transient stimuli and contact to prey (*t*-test, *P* = 0.574) between successful and non-successful approaches. Furthermore, we found no significant differences in the parameters onset of the approach phase (continuous cue: Mann–Whitney-*U*-test, *U* = 33.0, *P* = 0.536; transient cue: *t*-test, *P* = 0.620), end of approach phase (continuous cue: *t*-test, *P* = 0.578; transient cue: Mann–Whitney-*U*-test, *U* = 37.0, *P* = 0.791), minimal pulse duration at the end of the approach phase (continuous cue: *t*-test, *P* = 0.832; transient cue: *t*-test, *P* = 0.557), instant of QCF component elimination (continuous cue: *t*-test, *P* = 0.050; transient cue: *t*-test, *P* = 0.536), instant of first FM component reduction (continuous cue: *t*-test, *P* = 0.175; transient cue: Mann–Whitney-*U*-test, *U* = 42.0, *P* = 0.930), minimum pulse interval at the end of the approach phase (continuous cue: *t*-test, *P* = 0.285; transient cue: Mann–Whitney-*U*-test, *U* = 46.5, *P* = 0.625), first feet-water contact (continuous cue: *t*-test, *P* = 0.378; transient cue: *t*-test, *P* = 0.499), and last feet-water contact (continuous cue: *t*-test, *P* = 0.984; transient cue: *t*-test, *P* = 0.236). As we found no significant differences between successful and failed tasks in the mentioned parameters, we pooled the data and compared across tasks.

As expected, we found differences in the transient cue approach sequences compared to those with the continuous cue. The onset of the approach phase in continuous cue trials were rather stereotypic and began significantly earlier ($\bar{x}$ = −410 ± 79 ms, approximately −2.2 m) than in transient cue trials ($\bar{x}$ = −294 ± 105 ms, approximately −1.7 m) (*t*-test; *P* = 0.001). Variability was much lower in continuous cue trials (coefficient of variance = 19.3%) than in transient cue trials (coefficient of variance = 35.7%). The time difference between the onset of the transient cue and the instant of contact to prey was $\bar{x}$ = −466 ± 185 ms (range: −817 to −177 ms).

The reaction time, determined as the interval between the onset of the transient cue and the onset of the approach phase, was $\bar{x}$ = 171.5 ± 106.2 ms. Shortest reaction time was 50.9 ms.

The time difference between the end of the approach phase and the moment of prey contact was similar in both tasks (Mann–Whitney-*U*–Test, *U* = 174.0; *P* = 0.716). The feet were inserted into the water at similar instances across tasks as well (Mann–Whitney-*U*-test, *U* = 191.0, *P* = 0.367). Overall the feet were inserted into the water ($\bar{x}$ = −24.6 ± 9.8 ms) significantly after the end of the approach phase ($\bar{x}$ = −43.0 ± 14.6 ms) (*t*-test; *P* = 0.001) (Figure 4).

The reduction of overall pulse duration resulted in minimal values of $\bar{x}$ = 2.5 ± 0.4 ms at the end of the approach phase (Figure 4) and did not differ significantly across tasks (*t*-test, *P* = 0.083). The consistent decrease of pulse duration after the beginning of the approach phase mainly occurred in the QCF part of the call. It gradually disappeared while the FM component stayed largely unchanged. In continuous cue trials the QCF component was completely eliminated at −158 ms (median, quartiles 25%: −179 ms, 75%: −149 ms, approximately −0.9 m) before prey contact. The FM component remained stable in the approach phase until −149 ms (median, quartiles 25%: −173 ms, 75%: −132 ms, approximately −0.9 m) before prey contact. In transient cue approaches the QCF component was eliminated at $\bar{x}$ = −130 ± 36.1 ms (approximately −0.8 m) before contact, while the FM component remained unchanged until late in the approach phase at $\bar{x}$ = −144 ± 32.1 ms (approximately −0.9 m) before prey contact. The data for complete reduction of the QCF and FM components from transient cue trials may be affected by the random encounter of the regularly occurring transient cue stimuli by the bat.

Minimum pulse interval per trial was significantly shorter in continuous cue trials (median = 6.1 ms, quartiles 25%: 5.8 ms, 75%: 6.5 ms) than in transient cue trials (median = 6.8 ms, quartiles 25%: 6.1 ms, 75%: 7.1 ms) (Mann–Whitney-*U*-test, *U* = 233.0; *P* = 0.025) (Figure 4). Because of longer minimal pulse intervals in transient cue trials, final buzz 2 was significantly less frequent in transient cue trials (7 out of 18) than in continuous cue trials (13 out of 18) (Chi-square = 4.05, *P* = 0.0442).

### Prey contact

In all 36 trials the bats dipped at and passed the cue location within less than the span of a single foot. In reaction to the transient cue the bats dipped their feet at the location of the water splash while it was still occurring, or shortly after. Following successful spearing of prey that was hidden under the water surface, the bats proceeded to transfer it in flight from their feet to their mouths.

As expected, we found a significant difference in the dip duration between the two tasks, which lasted significantly longer during transient cue trials (median = 56.75 ms, quartiles 25%: 46.9 ms, 75%: 66.9 ms) than during continuous cue trials (median = 39.35 ms, quartiles 25%: 35.1 ms, 75%: 46.2 ms) (Mann–Whitney-*U*-test, *U* = 282.0, *P* = 0.001). A closer look revealed that dip duration was not significantly different up to prey contact (see above), but dip duration after prey contact was significantly longer in transient cue trials (median = 32.2 ms, quartiles 25%: 23.8 ms, 75%: 41.8 ms) compared to continuous cue trials (median = 13.4 ms, quartiles 25%: 10.8 ms, 75%: 15.5 ms) (Mann–Whitney-*U*-test, *U* = 320.0, *P* = 0.001).

### After prey contact

In both successful and failed capture attempts *N. leporinus* kept emitting echolocation calls after the end of the approach phase (Figure 2, calls between arrows 3 and 4, and shortly after arrow 4). Post buzz calls type 1 are emitted between the end of the approach phase and prey contact. Type 2 calls are emitted shortly before, during and shortly after prey contact.

Successful prey captures resulted in a pause of echolocation call emission while the bats transferred prey from their tail membrane to their mouths (Figure 3). In failed trials, the bats paused either only shortly in emission of echolocation calls or continued calling without pause (Figures 2, 3). In the latter we found a gradual transition from post buzz calls to orientation phase calls.

## Discussion

It has been known for some time that bats modify their echolocation behavior depending on the task on hand (e.g., Schnitzler et al., 2003; Holderied et al., 2008; Moss and Surlykke, 2010), but comparisons of echolocation behavior of a single bat species performing prey captures under different conditions remains scarce (Faure and Barclay, 1994), in particular in response to transient cues.

Here we compared the echolocation and dip performance of the trawling bat *Noctilio leporinus* when reacting to two different types of cues presented at a water surface. Trawling bat species may take continuously floating insects from the water surfaces but may also successfully attack transient targets, such as briefly surfacing small fish or crustaceans (Blood and Clark, 1998; Siemers et al., 2001; Aihartza et al., 2008). We asked whether the approach phase is a stereotypic behavior, or if it is specifically adapted to each cue suggesting a prey item, and focused on the similarities and differences of the bats' behavior across both tasks.

### Approach of the prey

As expected, all bats showed a clear approach phase in their echolocation behavior when coming closer to both types of cues, but we also found specific differences between the task-related echolocation behavior.

In continuous cue trials, we found a relatively stable onset of the approach phase at $\bar{x}$ = −410 ms/−2.2 m, whereas the onset of approach phase during transient cue trials occurred later ($\bar{x}$ = −294 ms/−1.7 m). The rather late onset of the acoustic approach phase in both tasks and the high sound pressure levels *Noctilio leporinus* uses in the field (max. 142.7 dB source level, Surlykke and Kalko, 2008), suggest a discrepancy between the distance of prey detection and the instant when the bats started to react to the cues indicating prey. The stimulus should have been detectable in the continuous cue trials at ca. 4.1 m distance from the prey (Stilz and Schnitzler, 2012, online calculator with the following settings: point reflector, dynamic range of 80 dB assuming a hearing threshold of 20 dB, 56 kHz, 26°C, and a humidity of 90%) with *N. leporinus* calling in our flight cage at a maximum intensity of 100 dB sound pressure level, measured 1 m before the mouth. Considering this calculated detection distance, *Noctilio* probably already detected the continuous cue while leaving the roost/perch but did not need to alter its echolocation behavior until −410 ms/−2.2 m before prey contact, a similar reaction distance as found for *Myotis lucifugus* avoiding wires (Grinnell and Griffin, 1958).

The difference in the values and variabilities of the approach phase onsets for the two tasks is influenced by the random onset of the transient cue stimulus relative to the bat's position. When exposing a free-flying bat to a transient cue we were not able to control for the bat's distance to the cue location. The transient cue water splash was triggered and the bat that was flying somewhere in the flight cage started to adapt its echolocation behavior when it was in a favorable position for cue detection. It is noteworthy that even in the six trials with stimulus onsets more than −410 ms before prey contact (Figure 3), we did not find approach phase onsets earlier than in the range of approach onset of continuous cue trials. In the remaining trials the transient stimuli had occurred close to or less than −410 ms before prey contact and a bat can only react after it perceives a stimulus, resulting in overall shorter approach phase onsets.

It is interesting that the shortest reaction time between stimulus onset and the onset of the approach phase was as short as 50.9 ms. This result corroborates a minimal reaction time of 47–63 ms for *Myotis nattereri* (Melcón et al., 2007).

### Call parameters during the approach

The differences in the call parameters during the approach phases of the two tasks were most likely due to the fact that the bats could steadily home in on the continuously detectable object (Surlykke et al., 2009), while the transient cue appeared and disappeared over time. In the continuous cue trials, all bats showed a rather stereotypic echolocation behavior, consisting of a stereotypical onset of approach phase and emission of final buzz I and in most cases also final buzz II, just as has been described for many aerial hawking bats prey captures (e.g., Pipistrelles and some vespertilionids: Schnitzler and Kalko, 2001; *Molossus molossus*: Mora et al., 2004). In contrast, our transient cue disappeared while bats were still approaching. The remaining circular waves on the water surface are unlikely to be perceived by the bats because they are non-breaking waves and therefore unlikely to be detectable through echolocation (Schnitzler et al., 1994). After the disappearance of the short-lived water splash, the bat changed its behavior from a typical approach to a prey object in a way similar to the echolocation behavior reported when *Myotis nattereri* approaches a landing site (Melcón et al., 2007). Similar to landing *M. nattereri*, our *N. leporinus* employed during the approach phase in transient cue trials longer pulse intervals, causing the final buzz II to be suppressed. Such prompt adaptations of call parameters to changes in conditions of the environment has also been reported for *Eptesicus fuscus* avoiding broadcast-echo ambiguity (Hiryu et al., 2010). We propose that bats, approaching a stable two dimensional water surface, without any specific object to home in on, require a lower information flow than aerial insect pursuit and capture with a prey object potentially moving in three dimensions (Schnitzler and Kalko, 2001; Melcón et al., 2007).

In contrast to the parameters mentioned above, the reduction in pulse duration, first by shortening the QCF component and only late in the approach phase by reduction of the FM component, is a stereotypical behavior (Schnitzler et al., 1994; Kalko et al., 1998). QCF components are adaptations that facilitate fluttering target detection (e.g., Schnitzler et al., 2003), detection of prey movement relative to the echolocating bat, and long distance detection of weak echoes (Simmons et al., 1975; Schnitzler and Kalko, 2001). Broadband FM components, in contrast, provide advantages for precise target localization (e.g., Schnitzler et al., 2003). Coming closer to a prey object at some point the bat enters the zone of pulse-echo overlap (e.g., Siemers and Schnitzler, 2000; Schnitzler and Kalko, 2001). As FM components are well suited for exact target localization at short distances, it is not surprising that the QCF component is reduced first (Schnitzler et al., 1994; Kalko et al., 1998). Assuming a speed of sound of 346.39 m/s at 25°C, an approximate end of the approach phase at $\bar{x}$ = −43 ms/approximately 35 cm distance to prey, and minimal pulse durations of 2.5 ms, the bats had entered the zone of pulse-echo-overlap shortly before the end of the approach phase. In earlier studies *N. leporinus* was found to enter the zone of pulse-echo overlap at a distance of 0.4 m from the prey (Hartley et al., 1989). For other species there are similar findings (*Eptesicus fuscus*: overlap in the last 60 ms or 18 cm, Wilson and Moss, 2004).

### Prey contact

In both tasks, the end of the approach phase occurred at $\bar{x}$ = −43.0 ms before prey contact (Figures 3, 4). We argue that at this point the bats had acquired all information needed for the capture attempt. Further calls, emitted shortly after the end of the approach phase but before, during, or shortly after prey contact, may serve a different purpose. Unlike other species (e.g., *Pipistrellus* sp.: Kalko, 1995; *Eptesicus fuscus* and *Myotis septentrionalis*: Wilson and Moss, 2004) that stop calling after the end of the approach phase, *N. leporinus* continues to emit post buzz calls type 1 and type 2 (Figure 2). Post buzz calls type 2 have previously been described in *N. leporinus* (Wenstrup and Suthers, 1984; Hartley et al., 1989).

Based on a reaction time of ~50 ms, the last part of final buzz II and post buzz calls type 1 and/or 2 occur so close to the time of prey contact that processing of new information and initiation of appropriate reactions would not be possible in time to serve for prey capture. Possibly the emission of these calls is a mechanism that ensures the availability of updated prey or environmental information after a failed capture attempt (Schnitzler and Kalko, 2001; Melcón et al., 2007; Ghose et al., 2009). Also, the increase in pulse amplitude in post buzz calls type 2 indicates a shift of attention from a close prey object to the bat's larger surroundings (Hartley et al., 1989). A similar shift of acoustic gaze before task completion has been observed in *Eptesicus fuscus* (Surlykke et al., 2009).

In our selected trials the water splash was vertical and the bats in our transient cue trials always dipped at the spot where the splash had occurred, suggesting the use of a spatial memory for prey capture (Moss and Surlykke, 2010). Interestingly, in some trials that were excluded from further analysis the water splash was not vertical but slanted, with the water hitting the surface at some distance from the “artifish” tube. In those cases the bats dipped up to several centimeters away from the location of the “artifish” near the point of water fall back to the surface (K. Übernickel, unpublished data). This indicates that the bats dip at the location of the cue latest in time, but this assumption would need further experiments.

### After prey contact

As expected, the bats' feet were inserted into the water at approximately the same point in time before anticipated prey contact in both tasks, but were dragged significantly longer through the water after passing the transient prey position than when reacting to the continuous cue. This behavior might illustrate the uncertainty of the bat about the submerged prey that is likely to be near the surface but at some distance from the initial position during the transient cue.

After a capture attempt, echolocation behavior continues in a differing manner, depending on hunting success or failure. While bats briefly ceased call emission after successful captures during the transfer of the prey into the mouth, this pause is considerably shorter in failed attempts (Britton and Jones, 1999), or may not be present at all (Figure 3). In extreme cases there is a gradual transition from post buzz calls to orientation phase calls, similar to the situation of an aborted buzz and the subsequent gradual transition back to search or early approach phase calls (Holderied et al., 2008).

## Conclusion

In conclusion, our results indicate that trawling bats possess the ability to modify their otherwise stereotyped echolocation behavior during approaches, within very short reaction times, depending on the sensory task. Even when an acoustic target disappears during an approach, they are still able to adapt their behavior and complete the task, dipping at the site of the transient cue based on spatial memory and dragging for a longer distance, presumably based on former experiences.

### Conflict of interest statement

The authors declare that the research was conducted in the absence of any commercial or financial relationships that could be construed as a potential conflict of interest.
